# Current knowledge of human Mpox viral infection among healthcare workers in Cameroon calls for capacity-strengthening for pandemic preparedness

**DOI:** 10.3389/fpubh.2024.1288139

**Published:** 2024-03-12

**Authors:** Alex Durand Nka, Yagai Bouba, Joseph Fokam, Aude Christelle Ka'e, Jeremiah Efakika Gabisa, Nadia Mandeng, Delors Jacques Toumansie Mfonkou, Chenwi Collins Ambe, Marie-Laure Mballa Mpouel, Tatiana Djikeussi, Boris Kevin Tchounga, Derrick Tambe Ayuk Ngwese, Debimeh Njume, Sonia Emmanuelle Mbala Nomo, Ezechiel Ngoufack Jagni Semengue, Armand Tiotsia Tsapi, Bernadette Bomgning Fokou, Ingrid Koster Simo Kamdem, Michel Carlos Tommo Tchouaket, Désiré Takou, Willy Pabo, Samuel Martin Sosso, Erick Tandi, Linda Esso, Georges Alain Etoundi Mballa, Anne-Cecile Zoung-Kanyi Bissek, Halle-Ekane Gregory Edie, Nicaise Ndembi, Vittorio Colizzi, Carlo-Federico Perno, Alexis Ndjolo

**Affiliations:** ^1^Chantal BIYA International Reference Centre for Research on HIV/AIDS Prevention and Management (CIRCB) Yaoundé, Cameroon; ^2^Faculty of Medicine and Surgery, University of Rome “Tor Vergata”, Rome, Italy; ^3^Faculty of Sciences and Technologies, Evangelical University of Cameroon, Bandjoun, Cameroon; ^4^Faculty of Medicine, UniCamillus - Saint Camillus International University of Health Sciences, Rome, Italy; ^5^National AIDS Control Committee, Central Technical Group, Ministry of Public Health, Yaoundé, Cameroon; ^6^Faculty of Health Sciences, University of Buea, Buea, Cameroon; ^7^National Public Health Emergency Operations Coordination Centre, Ministry of Public Health, Yaoundé, Cameroon; ^8^Faculty of Medicine and Biomedical Sciences, University of Yaoundé I, Yaoundé, Cameroon; ^9^Department of Health Biotechnology, Fobang Institutes for Innovations in Science and Technology, Yaoundé, Cameroon; ^10^Faculty of Health Sciences, University of Bamenda, Bamenda, Cameroon; ^11^Laboratory of Microbiology, University of Yaoundé I, Yaoundé, Cameroon; ^12^Elisabeth Glaser Peadiatric AIDS Foundation (EGPAF), Douala, Cameroon; ^13^Helen Keller International, Bafoussam, Cameroon; ^14^Faculty of Sciences, Department of Microbiology, University of Yaoundé I, Yaoundé, Cameroon; ^15^School of Health Sciences, Catholic University of Central Africa, Yaoundé, Cameroon; ^16^Faculty of Sciences, University of Buea, Buea, Cameroon; ^17^Faculty of Medicine, University of Antwerp, Antwerp, Belgium; ^18^Department of Disease, Epidemics, and Pandemics Control, Ministry of Public Health, Yaoundé, Cameroon; ^19^Division of Health Operational Research, Ministry of Public Health, Yaoundé, Cameroon; ^20^Africa Centres for Disease Control and Prevention, Addis Ababa, Ethiopia; ^21^Microbiology, IRCSS Bambino Gesu' Pediatric Hospital, Rome, Italy

**Keywords:** monkeypox (Mpox), knowledge, healthcare workers, emerging pathogens, Cameroon

## Abstract

**Introduction:**

An increased incidence of human Monkeypox (Mpox) cases was recently observed worldwide, including in Cameroon. To ensure efficient preparedness and interventions in the health system, we sought to assess the knowledge of Mpox's transmission, prevention, and response among healthcare workers (HCWs) in Cameroon.

**Methods:**

A cross-sectional online survey was conducted among HCWs in Cameroon using 21-item questions adapted from the United States Centers for Disease Control and Prevention (US-CDC) standard questionnaire on Mpox. The overall knowledge of Mpox was assessed by cumulative score and categorized as excellent (≥80%, 17/21) or good (≥70%, ≥15/21) knowledge. The regression analysis was used to identify the predictors of Mpox knowledge.

**Results:**

The survey enrolled 377 participants, but only responses from 342 participants were analyzed. Overall, 50.6% were female participants, and 59.6% aged 30 years or younger. The majority of the participants were medical doctors (50.3%); most worked in central-level hospitals (25.1%) and had 1–5 years of experience (70.7%). A total of up to 92.7% were aware of Mpox, with social media (58.7%) and radio/television (49.2%) as the main sources. The mean knowledge score was 14.0 ± 3.0 (4 to 20), with only 12.9% having excellent knowledge (≥80%) and 42.1% having good knowledge of Mpox. Younger age (26–30 years old) was associated with good knowledge, while workplace type was associated with excellent knowledge of Mpox (aOR [95% CI]: 4.01 [1.43–11.24]). Knowledge of treatment/management of Mpox was generally poor across the different professional categories.

**Conclusion:**

Knowledge of Mpox among HCWs is substandard across different professionals. Thus, for optimal preparedness and immediate interventions for Mpox and similar emerging pathogens, capacity-strengthening programs should be organized for HCWs while encouraging scientific literature and organizational social media websites.

## 1 Introduction

Human monkeypox (Mpox) is a viral zoonosis caused by the monkeypox (Mpox) virus belonging to the orthopoxvirus genus of the Poxviridae family (the same family as the virus that caused smallpox, which has now been eradicated). The virus is endemic in West and Central Africa, where it is thought to exist primarily in different types of rodents. There are two groups or “clades” of Mpox, one found in the Congo Basin of Central Africa with a case fatality of up to 10% and the other in West Africa with a case fatality rate of < 3% (1, 2).

Mpox can be transmitted via direct contact with infected body fluids, sexual contacts, lesion material from humans or animals, or indirect contact with contaminated material (3, 4). Human-to-human transmission occurs primarily through large respiratory droplets (5). The symptoms include fever, headache, malaise, muscle aches, swollen lymph nodes, and proctitis (6), followed by a rash a few days later that begins on the face and spreads to other parts of the body. The complications of monkeypox infections include secondary infections, bronchopneumonia, sepsis, encephalitis, and infection of the cornea with ensuing loss of vision. The illness can last up to 4 weeks but starts to fade when the skin lesions begin to subside (7). The virus is known to evade detection by the inhibition of the host antiviral immune response (antiviral chemokines, cytokines, and antigen presentation) and the suppression of the activation of T-cells (8).

Mpox was first identified in 1958 during an outbreak of Mpox in the Asian monkey *Macaca fascicularis*, which was used for polio vaccine research at an animal facility in Copenhagen, Denmark (9). The first Mpox case in humans was reported in the Democratic Republic of Congo (DRC, previously known as Zaire) in 1970, and the disease has remained endemic in the country and other African countries (2).

Since 2016, cases have appeared in the Central African Republic, Liberia, Nigeria, and Sierra Leone(1). In 2017, the largest outbreak of Mpox was reported in Nigeria, with 197 suspected cases and 68 confirmed cases, and by the end of 2018, the number of confirmed cases increased to 89, with a case fatality rate of 6.7% (1, 10). Human Mpox cases have also been previously reported in the United States in June 2003 (11, 12), in the UK (13) in September 2018, and in Israel (14) on 4 October 2018. In the case of the United States, Mpox was transmitted from infected native prairie dogs that were housed with infected exotic pets imported from Africa (11, 12), while in the UK (13) and Israel (14), patients were travelers who had returned from Nigeria.

As an epicenter or endemic country for Mpox, the Democratic Republic of the Congo conducts routine Mpox surveillance and clinical trials on potential Mpx vaccines among HCWs (15, 16). One of the important aspects of the surveillance system is to enhance the capacity of healthcare workers (HCWs) to identify and report cases and improve patient management (16). For an optimal response strategy, HCWs, particularly medical doctors and nurses, should have knowledge about the transmission patterns and clinical symptoms of Mpox to be able to quickly identify, report, and manage new cases to prevent further community-related or nosocomial transmission.

The Africa CDC outbreak brief on the MPox pandemic in January 2023 indicated that between January 2022 and January 2023, 1,296 cases and 228 deaths (CFR: 17.6%) in 13 African Union (AU) member states were reported. These countries include Cameroon (18 confirmed cases; 3 confirmed deaths), Benin (3 confirmed cases;0 confirmed deaths), Central African Republic (CAR) (13 confirmed cases;3 confirmed deaths), Congo (5 confirmed cases;3 confirmed deaths), the Democratic Republic of Congo (DRC) (319 confirmed cases;204 confirmed deaths), Ghana (116 confirmed cases;4 confirmed deaths), Liberia (6 confirmed cases;0 confirmed deaths), Nigeria (756 confirmed cases;7 confirmed deaths), Egypt (4 confirmed cases;0 confirmed deaths), Morocco (3 confirmed cases;0 confirmed deaths), Mozambique (1 confirmed cases;1 confirmed deaths), South Africa (5 confirmed cases;0 confirmed deaths), and Sudan (18 confirmed cases;1 confirmed deaths) (17).

In Cameroon, between 30 April and 30 May 2018, a total of 16 suspected cases (1 confirmed and 15 suspected cases) were reported to the Department of Disease, Epidemic and Pandemic Control of the Ministry of Public Health (18). These cases were identified in five health districts (HD) within five regions of Cameroon, namely, Njikwa HD (*n* = 6 suspected, *n* = 1 confirmed), Akwaya HD (*n* = 6 suspected), Biyem-Assi HD (*n* = 1 suspected), Bertoua HD (*n* = 1 suspected), and Fotokol HD (*n* = 1 suspected), with newer hot spots identified in other geographical locations, particularly, in the South West region (18). To mitigate this emerging global threat at the country level, the government of Cameroon developed and implemented a public health response strategy, which included the training of HCWs on infection prevention and control (IPC), with emphasis on the use of personal protective equipment, hand hygiene, and physical distancing, where necessary. Information related to the isolation of cases, symptomatic case management, and hand-washing techniques has been shared widely by the IPC workforce within hot spots and high-risk settings (19). A recent outbreak was reported in Cameroon in September 2022, in the South West region (20), and as of 19 April 2023, Cameroon had recorded 106 suspected cases, 18 confirmed cases, and 3 deaths related to Mpox (21, 22). These confirmed cases were found in four out of the five regions (South, Centre, North West, and South West), which called for the strengthening of the response strategy to stop its spread (23).

The increased number of human Mpox cases demonstrates the need and the importance of IPC, early detection, quick response, and the management of disease from HCWs. A report by the WHO and Africa CDC showed that one of the challenges faced in preventing the re-emergence of Mpox is the lack of sufficient knowledge about Mpox among HCWs in several countries, including high- and low-income settings (2).

Shafaati et al. (8) emphasized the importance of awareness and training campaigns to address the risks of sexual transmission of Mpox and prevent stigmatization of certain groups. A recent cross-sectional study assessing Mpox knowledge and attitudes of HCWs in some hospitals in Southern Italy in 2022 reported an unsatisfactory knowledge assessment, with a reported mean score of only 3.4 (0–9) (24). Furthermore, in a systematic review conducted by Mohamed L. and Abanoub A. in 2022, the overall knowledge of Mpox was unsatisfactory among nine articles, especially when assessing the knowledge of Mpox in Europe, the Middle East, and Asia (25). More precisely, poor knowledge of Mpox can lead to a large circulation of undiagnosed infections and thus skew epidemiological trends in resource-limited settings (RLS). Hence, in order to support the national response against Mpox at the country level, we sought to assess the knowledge of Mpox's transmission and management among HCWs in Cameroon.

## 2 Methods

### 2.1 Study design and settings

Within the framework of the country's response to Mpox, a cross-sectional online survey was conducted from August to October 2022 to assess the knowledge of Mpox viral infection among HCWs who are on service within the health system in Cameroon. The design and setting of this study were based on previous studies (26–28).

To achieve our intended goal, we used a random sampling method (self-administered online survey). According to Cameroon's Ministry of Public Health, the country has 39,720 health workers (29). Considering a 5% margin of error and a 95% confidence interval, a minimum of 381 participants were needed for this study. To ensure diversity, target participants, mainly medical doctors, nurses, and other HCWs (pharmacists, dermatologists, laboratory scientists, and nursing assistants) working at various levels of the healthcare system (central-level hospitals, district hospital (primary healthcare facilities), medicalized health centers, private hospitals, and other types of health facilities) were selected. The recruitment strategy involved reaching out to healthcare workers through social media, emails, and professional networks. Efforts were made to ensure diversity and representation by direct phone calls for participation and targeting underrepresented groups where necessary. We acknowledge that online surveys in Knowmedge, attitude and practice (KAP) studies are susceptible to some inherent biases including self-selection, non-response, social desirability, recall, sampling, access, and misinterpretation biases. These biases might have led to an unrepresentative sample, inaccurate responses, and underrepresentation of certain groups. To mitigate these potential biases, we used standardized assessment tools and provided clear instructions to minimize subjective interpretation. The Cameroonian health system has a National Public Health Emergency Coordination Centre with strategic and operational plans in response to infectious diseases of epidemics and pandemic potentials, including COVID-19, Cholera, Mpox, and viral hemorrhagic fevers. Field activities were conducted with the interventions of several stakeholders with a multi-sectorial approach in every hot spot and high-risk geographical location.

### 2.2 Survey instrument

A pre-tested and standard questionnaire was developed before the commencement of the study. The questionnaire consisted of questions to assess knowledge of Mpox and to collect a range of potential explanatory variables, with a total of 21 item multiple choice questions which were adapted from the United States Centers for Disease Control and Prevention (CDC) questionnaire (30) (see Appendix). The questionnaire was developed in both English and French, which are the two official languages of Cameroon. For maximal efficiency (validation), pre-testing (pilot) was performed among 20 independent HCWs who were not included in the study (10 medical doctors, 5 nurses, and 5 clinical laboratorians). The questionnaire was then finalized and validated using various feedback obtained from the pilot testing phase. After administering the survey with this pilot group of respondents and repeating the survey with the same group at a later point in time, there was a complete agreement (reliability) between the two time points (kappa = 1). The questionnaire content validity was approved by a majority of independent HCWs (90%, 18/20).

### 2.3 Data collection

Invitation to complete the anonymous online survey was sent using social media (mainly WhatsApp) or e-mails. Efforts were made to ensure the participation of HCWs from the rural areas, especially in the southern region where people were sensitized during meetings to take up the survey, and up to two reminders were sent after the initial message. The questionnaire entailed detailed features and contacts of the principal investigators for any further clarification, as well as the purpose of the study for informed consent prior to enrolment. The survey was estimated to take ~7–10 min to complete and without using any documentation. As the selection criteria, this study was limited to only active Cameroonian HCWs practicing in Cameroon, and those who were willing to participate and completed the questionnaire in ≤ 10 min without using any documentation were retained for analysis. The participants who fell short of the aforementioned requirements, as well as those who submitted incomplete responses, who submitted duplicate answers, with inconsistencies in their answers, and whose variables for assessing their level of knowledge were not clearly defined, were excluded from the study.

To ensure confidentiality, the names of the participants were not collected, and only the principal investigator had access to the survey account. At the end of the survey period, the raw data were extracted and imported into statistical software for analysis. Data were protected using specific anonymous and unique identifiers with a password-protected computer. To control and avoid resubmission, duplication, or multiple participation, we used unique identifiers such as email addresses or participant IDs. The study fulfilled the CHERRIES criteria (31).

### 2.4 Study variables

The response variable in this study was the knowledge of Mpox viral infection among HCWs in Cameroon. The questionnaire included knowledge of Mpox transmission, clinical features, and treatment/management. The questionnaire consisted of a 21-item questionnaire in which a correct response was scored one (1) and an incorrect response was scored zero (0). The scores were summed to give a total score ranging from 0 to 21. Two different cut-off scores were defined: ≥80% (at least 17/21) and ≥70% (at least 15/21), representing excellent and good knowledge of Mpox, respectively. Although previous studies used Bloom's cut-off point of 80–100% as good scores, 60–79% as moderate scores, and < 60% and below as poor scores (32), our team decided to create two subdivisions instead of three. Here, we chose to use two scenarios based on the 80% and 70% thresholds and considered scores < 70% as indicative of poor knowledge of Mpox. This decision was made to better distribute the survey's scores into more distinct categories given the volume of questions.

To facilitate the analysis and interpretation of data, we operationalized variables into specific categories and ranges. Four main groups of explanatory variables that could affect knowledge were categorized and assessed: sociodemographic characteristics, workplace characteristics, the characteristics of the medical specialty, and exposure to and/or sources of Mpox-related information. According to the distribution of participants, age was categorized into four specific ranges (20–25, 26–30, 31–39, and ≥40 years). The medical profession, defined as the completed/graduate medical or paramedical training, was grouped into the following: medical doctors, nurses, and other HCWs, which represent the three main categories of health workers in Cameroon. Workplace characteristics included the types of health facilities: central-level hospitals, district hospital (primary healthcare facilities), medicalized health centers, private hospitals, and other health facilities which represent the Ministry of Public Health's classification of health facilities. To assess the characteristics of the medical professionals, information on HCWs' job locations (rural or urban), their professional experiences (1–5, 6–10, 11–15, and ≥16 years), and whether they had attended any continuous education or training (local, national, and international conferences in the last 5 months) were collected. To assess exposure to or sources of Mpox-related information, the respondents were asked whether they had ever received Mpox information during their professional training and whether they had heard about Mpox prior to the interview. This categorization allowed for the capture of meaningful differences within these characteristics.

### 2.5 Statistical analysis

Frequencies, proportions, and confidence intervals were computed, and data were summarized using tables and figures. The associations between the explanatory variables and the dependent variables were assessed using a two-step logistic regression model for both ≥70% and ≥80% cut-off scores, representing good and excellent Mpox knowledge, respectively. Initially, all explanatory variables were analyzed separately in a univariate model, and variables with a *p*-value of ≤ *0.25* were then included in the multivariable logistic regression analysis to assess the impact of multiple independent variables on the likelihood of good knowledge of Mpox. Good knowledge of Mpox was the baseline variable used for comparison (outcome), and specific variables were chosen for inclusion based on their theoretical relevance to the outcome and existing evidence of their association with good knowledge of Mpox. For comparison, females were used as the reference for the “gender” variable; young HCWs aged 20–25 for the “age” variable, medical doctors for the “medical profession” category, the central hospital (tertiary healthcare facilities) for the “level of health facility”, and HCWs with 1–5 years of experience for “years of experience” category.

To ease result interpretations, the estimated crude odds ratio (OR) of unadjusted analyses and the adjusted OR (aOR) were interpreted in relation to a reference category. The significance was assessed at *p* = 0.05, and analyses were conducted using Statistical Package of Social Sciences version 22.0 software (SPSS Inc., Chicago, IL, USA).

### 2.6 Ethical considerations

In accordance with the Declaration of Helsinki on good clinical practices and ethical considerations, the present study was approved within the frame of multisectoral surveillance and in response to public health emergencies of zoonotic origin (authorization Ref. N° E2–168/L/MINSANTE/SG/DLMEP/SDLEP from the Ministry of Public Health in Cameroon). Prior to enrollment, the study information sheet was provided to each potential participant, and informed consent was then obtained from each participant. Data confidentiality and privacy of participants were ensured by the use of anonymized unique identifiers, and the data were secured in an encrypted password-protected computer. Only authorized individuals, such as the principal and co-principal investigators, had access to the survey account. The generated data were used to strengthen the capacity of the target population on better outbreak preparedness and response through result dissemination and exploitation.

## 3 Results

### 3.1 Respondents' characteristics

During the survey, a total number of 377 responses were received from study respondents, but 35 were excluded due to incomplete information and longer or shorter time of completing the questionnaire (i.e., < 5 min to mitigate the risk of bias or more than 15 min to limit events of answers following consultations of information from different sources before responding). Respondents were expected to complete the questionnaire between 7–10 min. The final analysis included 342 (90.7%) respondents, which represents ~90% (342/381) of the participation rate for the minimum sample size, with a margin of error of 5.3%. The characteristics of the surveyed HCWs are presented in Table 1.

**Table 1:** Factors associated with an excellent knowledge (80% threshold) of human Mpox infection among HCWs.

	**Good knowledge**		**Unadjusted**		**Adjusted**	
**Variables**	**Overall** ***N*** **(%)**	***n*** **(%)**	**OR (95% CI)**	***P*** **–value**	**aOR (95% CI)**	**P–value**
**Gender**						
Woman (R.)	173 (50.6)	19 (11.0)	1			
Man	169 (49.4)	25 (14.8)	1.40 (0.74–2.66)	0.294		
**Age group (year)**						
20–25 (R.)	48 (14.0)	2 (4.2)	1		1	
26–30	156 (45.6)	20 (12.8)	3.38 (1.20–5.40)	0.109	3.03 (0.66–13.83)	0.152
31–39	98 (28.7)	17 (17.3)	4.82 (1.00–4.60)	**0.041**	3.82 (0.75–19.39)	0.105
≥40	40 (11.7)	5 (12.5)	3.28 (0.60–17.94)	0.170	1.63 (0.17–15.27)	0.669
**Medical profession**						
Medical doctors (R.)	172 (50.3)	23 (13.4)	1		1	
Nurses	72 (21.1)	13 (18.1)	1.42 (0.67–3.00)	0.348	1.65 (0.85–3.18)	0.790
Others	98 (28.6)	8 (8.2)	0.57 (0.24–1.34)	0.201	0.32 (0.26–0.82)	**0.018**
**Level of health facility**						
Central hospital (tertiary healthcare facilities) level (R.)	86 (25.1)	7 (8.1)	1		1	
District hospital (primary healthcare facilities)	46 (13.5)	4 (8.7)	1.07 (0.29–3.88)	0.912	1.03 (0.27–3.81)	0.968
Medicalized health center	81 (11.1)	9 (11.1)	1.41 (0.50–3.98)	0.516	1.51 (0.51–4.42)	0.448
Private hospital	35 (23.7)	4 (11.4)	1.45 (0.39–5.32)	0.570	1.37 (0.36–5.17)	0.637
Others	94 (27.5)	20 (21.3)	3.05 (1.21–7.63)	**0.017**	4.01 (1.43–11.24)	**0.008**
**Years of experience**						
1–5 (R)	242 (70.8)	27 (11.2)	1		1	
6–10	48 (14.0)	10 (20.8)	2.09 (0.93–4.67)	0.071	1.49 (0.55–3.97)	0.426
11–15	31 (9.1)	5 (16.1)	1.53 (0.54–4.32)	0.421	1.49 (0.31–7.01)	0.613
≥16	21 (6.1)	2 (9.5)	0.83 (0.18–3.79)	0.819	1.01 (0.12–8.53)	0.993

p-values in bold indicate those that are statistically significant. The multivariable model was adjusted for age group, the medical profession, level of health facility, and years of experience. OR, odds ratio; aOR, adjusted odds ratio; CI, confidence Interval; R., reference category; HCW, healthcare workers.

Of the participants enrolled, 8 of the 10 regions of Cameroon were represented. More specifically, 42.6% (146/342) were from Yaounde, 11.9% (41/342) were from Douala, 10.8% were (37/342) from Bafoussam, 9.4% (32/342) were from Ngaoundere, 8.7% (30/342) were from Buea, 7.8% (27/342) were from Bertoua, 6.4% (22/342) were from Ebolowa, and 2.0% (7/342) were from Garoua. More than half of the participants, i.e., 172 (50.3%), were medical doctors. Concerning the gender of the participants, 50.6% (173/342) were female participants; for age, 59.6% were 30 years old or younger. Approximately 25.1% (86/342) of the respondents worked in central-level hospitals, 23.7% (81/342) in medicalized health centers, 10.2% (35/342) in private hospitals, and 27.5% (94/342) in other health facilities (research centers and non-governmental organization). Most of the HCWs (70.7%, 242/342) had a professional experience between 1 and 5 years (Table 1).

### 3.2 Source of information

**Figure 1:**
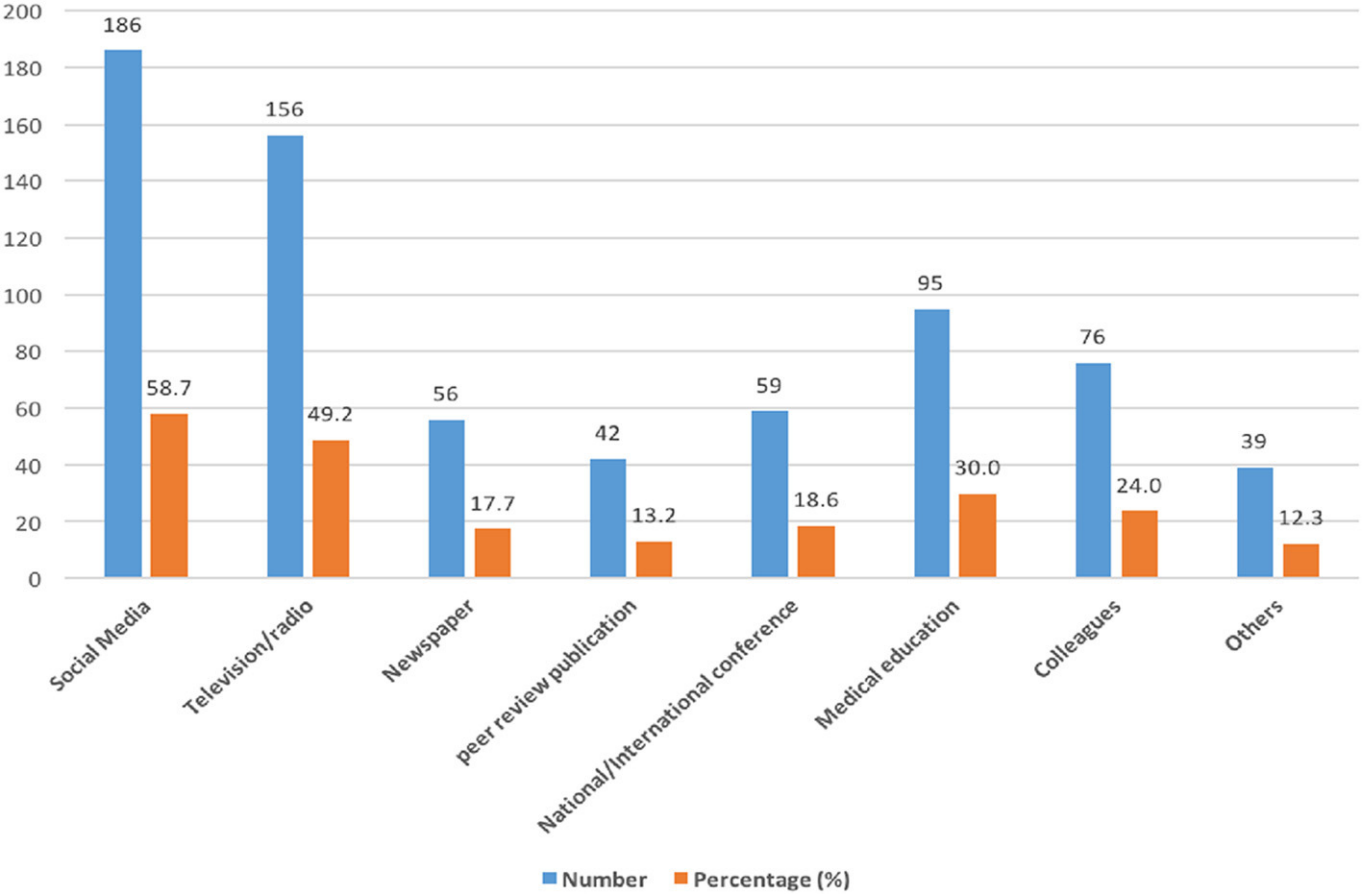
Sources of information on Mpox among HCWs. Some HCWs obtained Mpox information from multiple sources.

In this study, 92.7% (317/342) of the participants reported having heard about Mpox infection; of these, 58.7% (186/317) of them received their information from online media, and 49.2% (156/317) of them received their information from radio/television. Furthermore, 30% (95/317) of the participants gained their information during their medical training, 24% from colleagues, 13.2% from peer-review articles, 17.7% from newspapers or magazines, 18.6% from national or international conferences, and 12.3% from other sources (Figure 1).

### 3.3 Knowledge of mpox and associated determinants

The median score on Mpox knowledge was 14 (95% CI: 13–15), and the score ranged from 4 to 20. Using the 80% cut-off score, only 44 (12.8%) out of 342 respondents had an excellent knowledge of Mpox. When the cut-off was reduced to 70%, 42.1% (144 out of 342) of respondents had a good knowledge.

**Figure 2:**
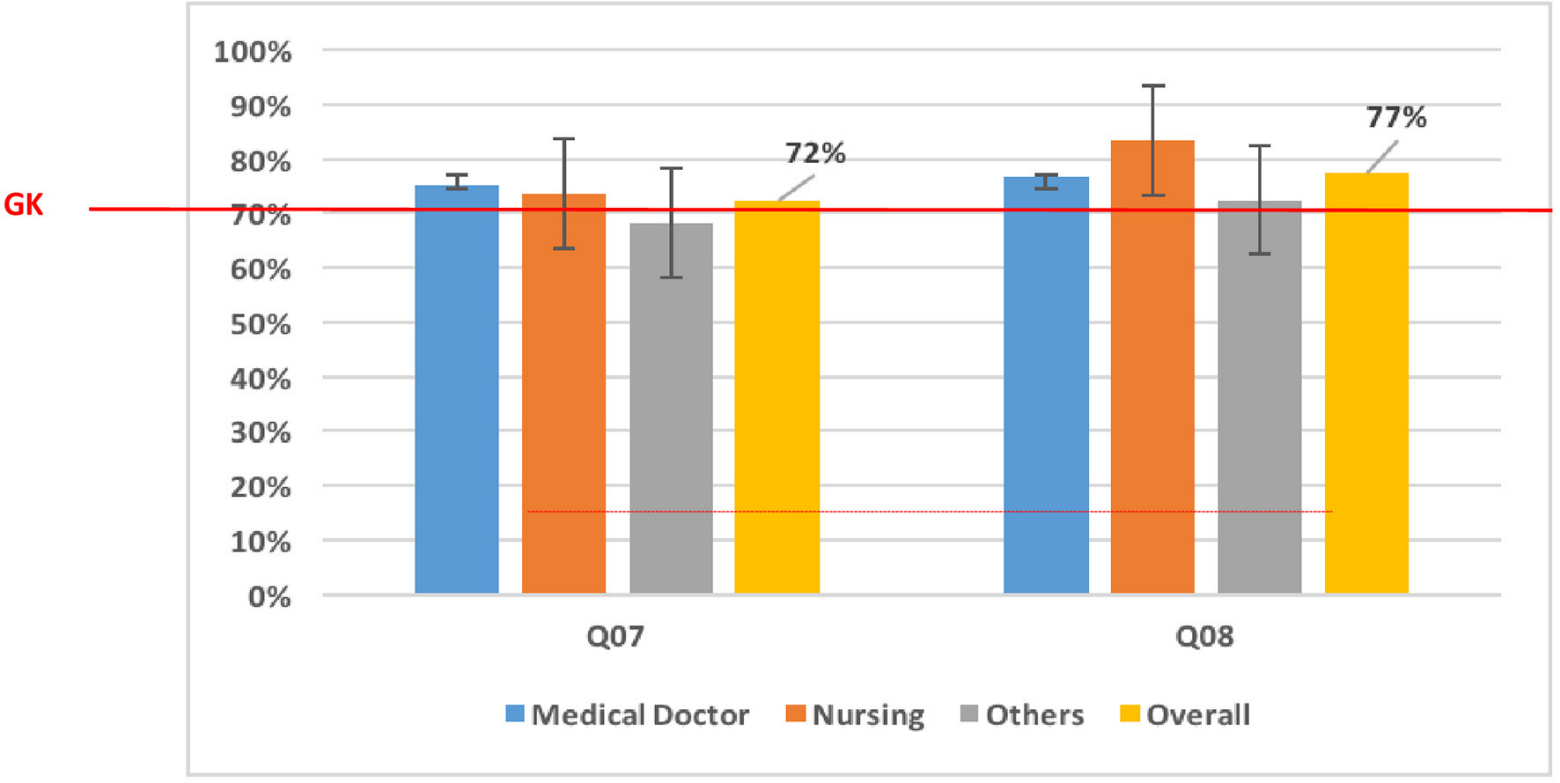
Knowledge of Mpox transmission means. “Others” (laboratory scientists, epidemiologists, pharmacy technicians, radiographers, physiotherapists, and dental technicians); “Overall” (mean of knowledge among medical doctors, nursing, and other categories); Q07 and Q08 represent questions 07 and 08 in the questionnaire used to assess the level of knowledge; Q07: Monkeypox is easily transmitted from human-to-human. Q08: Monkeypox could be transmitted through a bite of an infected monkey. GK, Good Knowledge (70% of good response).

**Figure 3:**
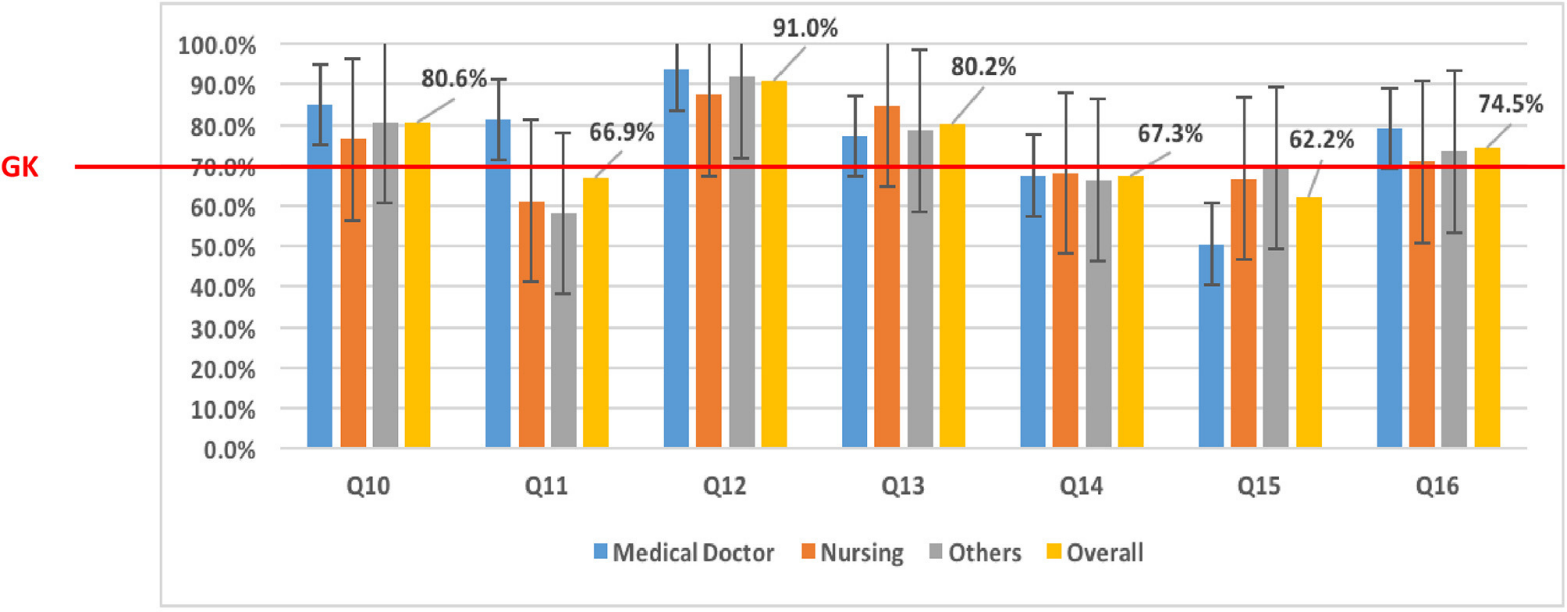
Knowledge of Mpox clinical diagnostics among HCWs. “Others” (laboratory scientists, epidemiologists, pharmacy technicians, radiographers, physiotherapists, and dental technicians); “Overall” (mean of knowledge among medical doctors, nurses, and Others categories); Q10, Q11, Q12, Q13, Q14, Q15, and Q16 represent questions 10 to 16 in the questionnaire used to assess the level of knowledge; Q10: Monkeypox and smallpox have similar signs and symptoms. Q11: Monkeypox and smallpox have the same signs and symptoms. Q12: Flu-like syndrome is one of the early signs or symptoms of human Monkeypox. Q13: Rashes on the skin are one of the signs or symptoms of human Monkeypox. Q14: Papules on the skin are one of the signs or symptoms of human Monkeypox. Q15: Vesicles on the skin are one of the signs or symptoms of human Monkeypox. Q16: Pustules on the skin are one of the signs or symptoms of human Monkeypox. GK, Good Knowledge (70% of good response).

**Figure 4:**
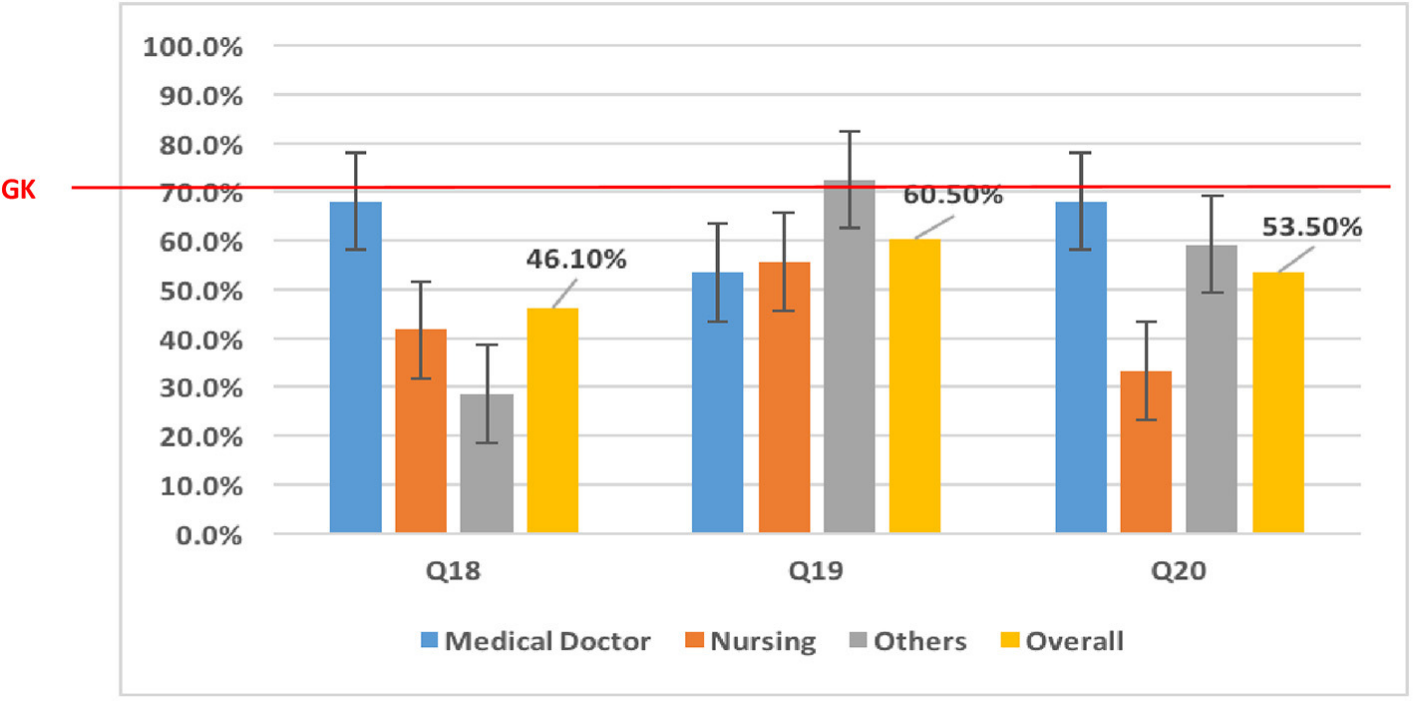
Knowledge on treatment/management of Mpox among HCWs. “Others” included laboratory scientists, epidemiologists, pharmacy technicians, radiographers, physiotherapists, and dental technicians. Q18, Q19, and Q20 represent questions 18 to 20 in the questionnaire used to assess the level of knowledge. Q18: One management option for patients with Monkeypox who are symptomatic is to use paracetamol. Q19: Antivirals are required in the management of human Monkeypox patients. Q20: Antibiotics are required in the management of human Monkeypox patients. GK, Good Knowledge (70% of good response).

Across some domains, the majority of the respondents had accurate knowledge of Mpox. For example, most (91.8%; 314/342) respondents stated that Mpox is caused by a virus, and more than 80% of them stated that Mpox and smallpox have similar signs and symptoms. Approximately 36.1% (218/342) of the respondents stated that some human Mpox cases were detected in Cameroon. Assessing respondents' “knowledge on[sic] transmission,” those in the “Others” category [68.3% (67/98)] had poor knowledge of human-to-human transmission (Figure 2). Concerning the zoonotic transmission of Mpox, the majority of participants had at least a good knowledge of ≥70% (Figure 2). Participant's knowledge of clinical features was generally good (≥70%) (Figure 3). However, no professional category had a good knowledge of the presence of vesicles and papules, which are key clinical features of Mpox (Figure 3). Knowledge of treatment/management was generally poor across the different professional categories (< 70%) (Figure 4).

The association of Mpox knowledge and some explanatory variables was assessed using both cutoff scores (i.e., 70% and 80%). Using the 80% cutoff score, at the univariate level, the age group of 31–39 years (17.3%) and the “Others” type of workplace were associated with excellent knowledge (OR:4.82 [95% CI:1.0–4.6s], *p* = 0.041; and OR:3.05 [95% CI:1.21–7.63], *p* = 0.017, respectively) compared to those aged 20–25 years and those who worked in central-level hospitals, respectively (Table 2). However, the multivariable analysis showed that the “Others” professional category (OR: 0.32 [95% CI: 0.26–0.82], *p* = 0.018) and the “Others” type of workplace category (OR: 4.01 [95% CI: 1.43–11.24], *p* = 0.008) were independently associated with excellent knowledge of Mpox.

**Table 2:** Factors associated with good knowledge (70% threshold) of human Mpox infection among HCWs.

			**Unadjusted**		**Adjusted**	
**Variables**	**Overall** ***N*** **(%)**	**Good knowledge** ***n*** **(%)**	**OR (95% CI)**	***P*** **–value**	**aOR (95% CI)**	***P*** **–value**
**Gender**						
Woman (R.)	173 (50.6)	76 (43.9)	1			
Man	169 (49.4)	68 (40.2)	0.78 (0.45–1.35)	0.388		
**Age group (year)**						
20–25 (R.)	48 (14.0)	12 (25.0)	1		1	
26–30	156 (45.6)	73 (46.6)	2.63 (1.20–5.40)	**0.009**	2.74 (1.29–5.80)	**0.008**
31–39	98 (28.7)	41(41.8)	2.10 (1.00–4.60)	**0.049**	1.96 (0.84–4.54)	0.117
≥40	40 (11.7)	18 (45.0)	2.40 (0.90–6.10)	0.051	2.69 (0.68–10.58)	0.155
**Medical profession**						
Medical doctors (R.)	172 (50.3)	64 (37.2)	1		1	
Nurses	72 (21.1)	36 (50.0)	1.68 (0.96−2.94)	0.065	1.65 (0.85–3.18)	0.136
Others	98 (28.7)	44 (44.8)	1.37 (0.83–2.27)	0.216	1.42 (0.81–2.49)	0.218
**Level of health facility**						
Central hospital (tertiary healthcare facilities) (R.)	86 (25.1)	30 (34.9)	1			
District hospital (primary healthcare facilities)	46 (13.5)	18 (39.1)	1.09 (0.45–2.59)	0.844		
District medical centers (primary healthcare facilities)	35 (10.2)	31 (38.3)	2.89 (0.90–9.30)	0.751		
Private hospital	81 (23.7)	20 (57.1)	1.35 (0.65–2.83)	0.416		
Others	94 (27.5)	45 (47.9)	1.53 (0.70–3.34)	0.286		
**Years of experience**						
1–5 (R)	242 (70.8)	101 (41.7)	1			
6–10	48 (14.0)	22 (45.8)	1.18 (0.63–2.20)	0.600		
11–15	31 (9.1)	13 (41.9)	1.00 (0.47–2.15)	0.983		
≥16	21 (6.1)	8 (38.1)	0.85 (0.34–2.14)	0.745		

p-values in bold indicate those that are statistically significant. The multivariable model was adjusted for age group and medical profession. OR, odds ratio; aOR, adjusted odds ratio; CI, confidence Interval; R., reference category; HCW, healthcare workers.

With the lower cut-off score (70%), the age groups 26–30 (46.6%) and 31–39 years (41.8%) were associated with good knowledge of Mpox (OR: 2.63 [95% CI: 1.20–5.40], *p* = 0.009; and OR: 2.1 [95% CI: 1.0–4.6], *p* = 0.04, respectively), when compared to those aged 20–25 years. However, in the multivariate analysis, only the age group 26–30 years was associated with a good knowledge of Mpox (OR: 2.74; 95% [CI: 1.2–5.8], *p* = 0.008) when compared to the age group 20–25 years.

## 4 Discussion

Responding to outbreaks, such as Mpox, requires a strong collaboration between all stakeholders, including frontline healthcare workers. In Cameroon, both event-based and case-based surveillance are put in place, but the current surveillance system mainly relies on case-based surveillance. Therefore, it is paramount that HCWs (particularly medical doctors and nurses) get a good mastery of the knowledge and case definitions and the management of potential epidemic diseases. This is because they are responsible for the early detection and management of cases at health facility levels. For this reason, our study aimed to assess the knowledge of HCWs in Cameroon on the ongoing Mpox infection, considering the transmission, clinical features, and management/treatment of the infection.

Data generated from this study revealed that, in general, the knowledge of HCWs on Mpox in Cameroon was poor (42%). Less than 15% of the participants were able to answer correctly to 80% of the 21 questions. When looking at some of the factors associated with knowledge of Mpox at an 80% cut-off score, we found that HCWs other than medical doctors and nurses had especially poor knowledge of Mpox. It was worrisome to observe that < 20% of medical doctors and nurses recorded an excellent understanding because they are directly involved with patient care.

It was interesting to note that those in the categories of other health facility levels, including research centers and non-governmental organizations (NGOs), showed a slightly higher knowledge than those in hospital settings, which might be partly justified by the fact that several respondents in this category are involved with the design or implementation of public health policies related to the Mpox response. It was, for example, reported that public health NGOs have specific missions, with most largely embodying epidemiological surveillance of infectious diseases, which perhaps exposes them more to new emerging and re-emerging health conditions (33). The other variables including age, gender, and the number of years of work experience did not seem to show a significant difference in the Mpox knowledge. This finding indicates a uniformly low level of Mpox knowledge across these variables. This low knowledge of Mpox among HCWs is not only limited to Cameroon, as a previous study found a uniformly low knowledge among general practitioners in Indonesia (34). Moreover, a cross-sectional study conducted in 2022 to assess the knowledge and attitudes of HCWs in some hospitals in Southern Italy reported unsatisfactory knowledge (24). A systematic review by Mohamed L. and Abanoub A. showed that the overall understanding of Mpox was poor among nine articles, which exclusively assessed Mpox knowledge in Europe, the Middle East, and Asia (25). As Mpox was a rare disease, it received less attention. The recent pandemic of Mpox spread faster at a large scale and affected the most vulnerable populations, therefore indicating that more attention should be given to it.

In the present study, even at a threshold of 70% (here referred to as good knowledge), < 50% of the participants had good knowledge. Most of the participants, including medical doctors, had poor knowledge (< 70%) of the evolution and presentation of the classic clinical features of Mpox and case management. It should be noted that most of the HCWs who participated in this study were still in their early career, with only 1–5 years of working experience, which could have impacted their poor knowledge.

An exploratory analysis based on the cut-off score knowledge of 70% was equally carried out. A multivariable analysis indicated that those aged 26–30 years had a higher level of knowledge (47%) than those in other age groups. The age group of 26–30 years is part of the social media-friendly group; consequently, they might be more likely to get Mpox-related information. Of note, ~58% of the participants reported receiving information about Mpox via online media platforms (Facebook, WhatsApp, podcast, etc). It was reported elsewhere that young HCWs tend to prefer to consult social media networks for information because of their rapid accessibility (35). Despite some information lacking validity, social networks have the particularity of transmitting data in record time and with a larger coverage. In this digital era, social media can represent an effective communication channel that can provide continuous education to HCWs (36). There was uniformly low knowledge of Mpox, considering other variables such as gender, type of workplace, work experience, and medical training. This finding suggests that, in such a context, the infection can spread unnoticed in the community without being detected/reported timeously. Therefore, strategies for enhancing the knowledge of HCWs on the detection and management of zoonotic Mpox are needed, including sensitization of HCWs via online platforms to respond adequately to such outbreaks (37). These strategies are particularly important as they resonate with the One Health approach for sustainable infection prevention and control (38).

In the frame of pandemic preparedness and interventions, considering the reported pitfalls among HCWs would guide global health agencies (WHO, Africa CDC, etc) in tailoring capacity-building or strengthening programs for optimal efficiency in epidemic/pandemic preparedness and response at the continental and global levels.

This study has some limitations. This was an online survey that required an internet connection; as such, there was a potential selection bias in relation to the availability of internet access, especially in rural areas (39).

## 5 Implications and recommendations

The study's findings highlight the critical need for targeted training programs to enhance healthcare workers' (HCWs) understanding of epidemic diseases, such as Mpox, particularly among medical doctors and nurses. The uniformly low level of Mpox knowledge across various demographic and professional variables highlights the potential impact on outbreak response and the urgent need for comprehensive capacity-building efforts. To address these challenges, it is recommended to leverage coordinated social media and online platforms for continuous education and sensitization of HCWs, considering their accessibility and potential to reach a wider audience. In addition, there is a need to conduct representative studies to ensure a comprehensive understanding of HCWs' knowledge levels nationwide (to overcome potential selection biases related to internet access, especially in rural areas), thereby guiding the development of capacity-building initiatives and pandemic preparedness strategies. These implications and recommendations are crucial for guiding the development of capacity-building initiatives and pandemic preparedness strategies at both national and global levels.

## 6 Conclusion

Knowledge of Mpox among HCWs within the health system of Cameroon is uniformly low across sociodemographic, workplace, and medical professional characteristics. Thus, for optimal preparedness and interventions on IPC, case management, and surveillance of Mpox and similar emerging pathogens, capacity-strengthening programs should be reinforced in the Cameroonian context and similar settings, with a particular focus on HCWs in clinical facilities and the older adults, while encouraging scientific literature and organizational social media web sites. Such evidence-based interventions could also support response in several African settings.

## Data availability statement

The raw data supporting the conclusions of this article will be made available by the authors, without undue reservation.

## Ethics statement

Ethical review and approval was not required for the study on human participants in accordance with the local legislation and institutional requirements. Written informed consent from the participants was not required to participate in this study in accordance with the national legislation and the institutional requirements. In accordance with the Declaration of Helsinki on good clinical practices and ethical considerations, the present study was approved within the frame of multisectoral surveillance and in response to public health emergencies of zoonotic origin (authorization Ref. N° E268/L/MINSANTE/SG/DLMEP/SDLEP from the Ministry of Public Health in Cameroon). Prior to enrollment, the study information sheet was provided to each potential participant, and informed consent was then obtained from each participant. Data confidentiality and privacy of participants were ensured by the use of anonymized unique identifiers, and the data were secured in an encrypted passwordprotected computer. Only authorized individuals, such as the principal and co-principal investigators, had access to the survey account. The generated data were used to strengthen the capacity of the target population on better outbreak preparedness and response through result dissemination and exploitation.

## Author contributions

ADN: Conceptualization, Data curation, Formal analysis, Writing—review & editing. YB: Data curation, Methodology, Writing—original draft, Writing—review & editing, Validation. JF: Conceptualization, Data curation, Formal analysis, Supervision, Validation, Visualization, Writing—original draft, Writing—review & editing. AK: Data curation, Investigation, Software, Writing—original draft. JG: Conceptualization, Formal analysis, Writing—review & editing. NM: Methodology, Project administration, Software, Supervision, Writing—original draft. DM: Formal analysis, Investigation, Methodology, Writing—review & editing. CA: Conceptualization, Formal analysis, Methodology, Visualization, Writing—review & editing. M-LM: Conceptualization, Data curation, Investigation, Visualization, Writing—review & editing. TD: Conceptualization, Data curation, Formal analysis, Writing—review & editing. BT: Data curation, Formal analysis, Project administration, Software, Writing—review & editing. DA: Data curation, Investigation, Resources, Writing—original draft. DN: Investigation, Writing—review & editing. SM: Investigation, Writing—review & editing. EN: Data curation, Formal analysis, Investigation, Writing—review & editing. AT: Investigation, Writing—review & editing. BF: Investigation, Writing—review & editing. IS: Investigation, Writing—review & editing. MT: Investigation, Writing—review & editing. DT: Investigation, Writing—review & editing. WP: Investigation, Writing—review & editing. SS: Funding acquisition, Investigation, Writing—review & editing. ET: Formal analysis, Writing—original draft, Writing—review & editing. LE: Investigation, Writing—review & editing. GE: Investigation, Writing—review & editing. A-CZ-K: Investigation, Writing—review & editing. H-EG: Investigation, Writing—review & editing. NN: Data curation, Methodology, Writing—original draft, Writing—review & editing. VC: Data curation, Writing—original draft, Writing—review & editing. C-FP: Conceptualization, Data curation, Writing—original draft, Writing—review & editing. AN: Writing—original draft, Writing—review & editing.

## Appendix

Questions used to measure the knowledge of monkeypox among general practitioners in Indonesia

Questions used to measure knowledge

No. Question Yes No

1 Monkeypox is prevalent in Southeast Asia countries

2 Monkeypox is prevalent in Western and Central Africa

3 There are many human monkeypox cases in Cameroon (greater than 10 cases)

4 There is an outbreak of human monkeypox in the center region of Cameroon

5 Monkeypox is a viral disease infection

6 Monkeypox is a bacterial disease infection

7 Monkeypox is easily transmitted human-to-human

8 Monkeypox could be transmitted through a bite of an infected monkey

9 Travelers from America continent are the main source of imported cases of monkeypox

10 Monkeypox and smallpox have similar signs and symptoms

11 Monkeypox and smallpox have the same signs and symptoms

12 Flu-like syndrome is one of the early signs or symptoms of human monkeypox

13 Rashes on the skin are one of the signs or symptoms of human monkeypox

14 Papules on the skin are one of the signs or symptoms of human monkeypox

15 Vesicles on the skin are one of the signs or symptoms of human monkeypox

16 Pustules on the skin are one of the signs or symptoms of human monkeypox

17 Lymphadenopathy (swollen lymph nodes) is one clinical sign or symptom that could be used to differentiate monkeypox and smallpox cases

18 One management option for monkeypox patients who are symptomatic is to use paracetamol

19 Antivirals are required in the management of human monkeypox patients

20 Antibiotics are required in the management of human monkeypox patients

21 Diarrhea is one of the signs or symptoms of human monkeypox
